# Analysis of collapse risks under cut and cover method based on multi-state fuzzy Bayesian network

**DOI:** 10.1371/journal.pone.0321382

**Published:** 2025-05-07

**Authors:** Ping Liu, Xueqiang Jin, Yongtao Shang, Jiaolan Zhu

**Affiliations:** 1 School of Civil Engineering, Lanzhou University of Technology, Lanzhou, China; 2 School of Management Science & Real Estate, Chongqing University, Chongqing, China; Southwest Petroleum University, CHINA

## Abstract

The collapse accidents under cut and cover method in metro station construction occurred frequently, leading to severe casualties and property damage. With increasing of metro station construction in China, more and more attention has been paid to collapse under cut and cover method. However, this subject is still not well studied and understood in China. To fill the research gap, this paper investigates collapse risk in the cut and cover method construction process using a multi-state fuzzy Bayesian network. Firstly, based on accident statistical analysis, 9 intermediate factors and 16 bottom factors of collapse were identified, and then a multi-state Fuzzy Bayesian network model was established based on these causative factors. Secondly, triangular fuzzy functions were utilized to fuzzily the data of nodes, and conditional probabilities were used to represent the uncertainty relationship between nodes. Additionally, an expert credibility-based survey method was employed to ensure the accuracy of node failure probability assessment. The method was applied to predict the risk of a case project using cut and cover method, and the results demonstrated that the probabilities of no-failure, moderate-failure, and severe-failure were 71%, 19%, and 10%, respectively. Sensitivity analyses of multi-states were performed to identify the key causal factors for moderate and severe collapse. The method can be used to predict the risk probability and key causal factors for collapse accidents. The result can provide decision support for cut and cover method construction, which could contribute to reducing the occurrence of collapse.

## 1. Introduction

Safety accidents in the construction industry occurred frequently, resulting in serious injuries and property damage. In the UK, with 45 casualties in 2022, the construction industry has the highest frequency of accidents [1]. The US construction industry has much higher fatalities and injuries than other industries, with 951 occupational fatalities in 2021 [2]. In China, there were 689 safety accidents and 794 fatalities in the construction industry in 2020 [3]. These statistics show that many countries face challenges in safety management of construction industry. Metro engineering, as a branch of the construction industry, has grown rapidly in recent years. According to the China Association of Metro [4], 55 cities have opened urban railways by the end of 2022, with a total mileage of 10,287.45 km, and the planned and under-construction projects still show a growing trend.

The majority of metro station projects are conducted underground, and the main construction method used is Cut and Cover Method (CCM). CCM refers to excavating foundation pits or trenches from the surface downwards. After excavation to the design elevation, construction is carried out from the bottom of the pit upwards. Finally, the earth is backfilled after the main structure of the underground work is completed. CCM is widely used in metro station construction due to geological conditions, short construction duration, and low cost. According to the statistics, underground works constructed by CCM account for more than two-thirds of the total number of soft soil works [5]. As a commonly used construction method, CCM has unique advantages in building underground projects. However, the construction process is susceptible to collapse accidents caused by many factors, such as engineering geological conditions, ground environmental conditions, support methods, excavation scale, etc. [6]. Liu et al. [7] counted 217 metro construction accidents from 2003 to 2022, and CCM accounted for 29% of the accidents, while COLLAPSE accidents were the most common type of accidents in CCM. collapse often causes mass casualties and triggers a chain of other disasters, resulting in significant property damage and casualties. For instance, the collapse of metro station construction in Hangzhou in 2008, resulted in 21 fatalities, 24 injuries, and a direct economic loss of 49.61 million dollars [8].

The risk of collapse in metro construction has received widespread attention. Compared with other accident types, collapse accidents cause more serious casualties and property losses [9]. Depending on the construction environment, collapse accidents can be categorized into various types, such as tunnel collapse, collapse, building collapse, and scaffolding collapse, etc. In the research on tunnel collapse, Zhang et al. [10] proposed a probabilistic evaluation method for tunnel collapse risk, which analyzed the causative factors of tunnel collapse accidents of various scales. Zhang et al. [11] utilized Bayesian networks (BN) to analyze the relationships among influencing factors in tunnel collapse incidents, and rank the factors influencing the risk of tunnel collapse. Huang et al. [12] proposed a dynamic assessment method for collapse risk, which can be used to calculate the risk’s indicator weights. In the study of the collapse risk of CCM, only a handful of studies focused on the risk analysis of CCM. However, it is generally evaluated qualitatively by design, construction and supervision, relying on past engineering experiences. This approach aims to identify the possible risk incidents during the excavation process of the station and propose macro-level response strategies. Sometimes, the risk analysis of CCM is also evaluated qualitatively based on factors such as site excavation and support, monitoring and management. For example, Li et al. [13] quantitatively evaluated the collapse risk based on the factors within the affected area. Graham et al. [14] studied the safety risks and failure mechanisms of deep foundation pit support. However, these risk assessment methods often lag behind accidents and fail to achieve the goals of risk inference and proactive control. Besides, traditional methods for risk analysis of such complex systems with multi states have limitations. The traditional “two-state system” describes the failure state of the system in terms of “occurrence” and “non-occurrence”. Soussa [15] introduced a model that assesses the geological risk of tunnels based on this “two-state system”. Similarly, Zhang et al. [11] proposed a decision-making method for tunnel collapse risk utilizing “two-state system”. However, there are multiple failure states in actual projects, and the use of the traditional “two-state system” to describe the failure state of the system has some inaccuracies and does not accurately reflect the actual failure state of the system.

To achieve risk inference and proactive control, some scholars have utilized Fault Tree Analysis (FTA) and Bayesian Networks (BN) for metro risk analysis. FTA is mainly used for qualitative risk analysis and prediction in complex projects [16]. Hyun et al. [17] applied FTA to study the risks during tunnel piercing. However, FTA has limitations in dealing with probability dependencies and uncertainties due to its reliance on static structures and uncertainty handling. Yang et al. [18] argued that FTA is susceptible to subjective factors and cannot accurately reflect the characteristics of projects under parameter changes. In comparison to FTA, BN has the advantage of explicitly representing the dependencies between events and enables real-time accident analysis. Sousa et al. utilized BN models to predict soil changes during metro tunnel construction. Sun [19] and Li [20] et al. used BN models to assess and predict collapse risks in tunnel engineering. Khakzad et al. [21] proposed a BN-based safety analysis method in process facilities. However, the previous studies require exact node failure probabilities for accident analysis.

In the metro construction industry, obtaining the exact value of nodal failure probability is difficult due to the lack of accurate accident data. Additionally, the patterns and mechanisms of FPSA are complex and diverse, resulting in fuzziness and uncertainty in the failure probabilities. Gong et al. [22] pointed out that reasoning and predictions based on uncertain data also lead to uncertain results, making it challenging to provide decision-making guidance. Hanss [23] highlighted that fuzzy set theory (FST) is an effective means to deal with uncertainty problems. Gong et al. [24] used FST to study the stability of slopes. Zhang et al. [11] proposed an improved BN model by integrating FST with BN to assess the collapse risk probability of a metro project. Subsequently, Zhang et al. [10] proposed a Fuzzy Bayesian Network (FBN) tunnel collapse risk evaluation model based on expert credibility. Zhang et al. [25] utilized FBN to analyze the risk of adjacent pipeline damage in tunnel engineering, providing theoretical support for safe tunnel construction. Therefore, FBN serves as an effective approach to incorporating uncertain factors into probabilistic analysis, enabling inference and prediction when data are incomplete or difficult to obtain.

In the existing studies, more and more attention has been paid to collapse under CCM. Nevertheless, collapse risks under cut and cover method have not well studied and understood. Differences in the environment and construction processes lead to variations in the risk factors and outcomes of collapses in various construction methods. Emphasizing the distinctiveness of construction methods in collapse risk management can contribute to control of collapse risks. According to Wang et al, collapse accidents accounted for about 39.09% of metro construction accidents and 39.75% of CCM construction accidents [26]. Compared to collapse risks in other construction methods, collapse in CCM is more frequent and often result in significant losses [6]. Therefore, this paper aims to analyze the probability and risk factors of collapse in CCM to provide decision support for the analysis of collapse incidents.

The following are the three objectives to achieve the research aim:

(1) identify causative factors of collapse accidents during CCM construction process
(2) establish analytical and predictive modeling of collapse accidents
(3) evaluate the probability of collapse risk and identification of key causal factors

## 2. Literature review

### 2.1. Risk management of CCM

Regarding in risk management for CCM, Faber et al. [27] proposed a risk pre-control model to study the main risk factors and risk evolution paths. Yin et al. [28] investigated the safety risks and failure mechanisms of deep foundation pit support structures during CCM construction. Choi et al. [29] proposed a standardized risk evaluation method that includes risk identification, risk analysis and risk evaluation. In terms of risk identification, Yu et al. [30] identified safety attitude, construction management and government supervision as the most important factors affecting metro safety. Zhang et al. [31] found that imperfect management was the biggest accident causative factor and most serious accidents were caused by pipeline leakage and poor geological conditions.

The risk analysis and evaluation of CCM mainly consists of qualitative and quantitative methods. Regarding qualitative method, Zou et al. [6] established a risk assessment checklist through literature research and expert interviews. By interviews with safety management experts, Zhang et al. [31] pointed out that the leadership team should develop a positive safety attitude. Ding et al. [32] established a safety risk identification system for pre-construction risk assessment. Those researches generally evaluated qualitatively by design, construction and supervision, relying on past engineering experiences. Sometimes, the risk analysis of CCM is also evaluated qualitatively based on factors such as site excavation and support, monitoring and management. For example, Graham et al. [14] studied the safety risks and failure mechanisms of deep foundation pit support. Li et al. [13] quantitatively evaluated the collapse risk based on the factors within the affected area. Tavallaie et al. [33] employed a finite element analysis method to assess the damage risk of the surrounding buildings during CCM. Zhou et al. [34] established a metro safety database containing accidents, attempted accidents and unsafe behaviours, which is used as a quantitative tool for assessing safety risks.

### 2.2. Risk management of collapses

Most existing studies analyzed collapse accidents following the general risk management process, including risk identification, analysis, assessment and control. Risk identification is a key step in risk management, aiming to identify the causes of risks. Zou and Li [6] used a risk questionnaire and Fuzzy Analytic Hierarchy Process to identify a list of safety risks. Zhou et al. [35] used accident cases to establish a metro construction collapse risk propagation path diagram. They obtained the factors affecting the collapse risk regarding personnel, environmental machinery, etc. Zhou [36] pointed out that personnel, environment and machinery are the direct factors affecting the impact of collapses, while management is an indirect factor. Shan et al. [37] used finite element analysis to illustrate that load distribution, resistance and damage modes significantly affect frame collapse accidents. Zhang et al. [10] found that the support method is critical to prevent collapse. Gong et al. [24] concluded that collapses are mostly related to site drainage and trapping.

In terms of risk analysis and assessment of collapse, Julie and Debra [38] proposed a pre-construction risk assessment procedure to minimize the impact of collapses on adjacent buildings. Fang et al. [39] utilized FTA model to investigate the chain of major collapse accidents and the evolutionary path of the Guangzhou metro. They found that risk assessment and investigation, safety awareness, and behavioral habits are critical to preventing and controlling collapse risk. Zhou et al. [40] used twin-digital technology to analyze the potential risk mechanism of collapse accidents to improve the digital prevention and control of risk. Risk control is the ultimate aspect of risk management, which aims to reduce losses resulting from risks. Quintana and Camet [41] utilized accident prediction modeling of potential hazard sources to improve safety performance. Meng et al. [42] compared risk control measures in different situations to select more effective risk control measures. To mitigate safety risks in metro stations, sensor technology was applied for real-time control of risks [43].

### 2.3. FBN

Fault tree analysis (FTA) and BN are widely used to qualitative risk analysis and prediction in complex projects [16]. Hyun et al. [17] applied FTA to study the risks during tunnel piercing. However, the FTA’s static structure makes it have limitations in dealing with probability dependencies and uncertainties. Yang et al. [18] argued that FTA is susceptible to subjective factors and cannot accurately reflect the characteristics of projects under parameter changes. Compared to FTA, BN has the advantage of explicitly representing the dependencies between events and enables real-time accident analysis. Sousa et al. [15] predicted the risk of metro tunnel construction by using BN, which provided decision-makers with a construction strategy that minimized the risk. Sun [19] and Li [20] used BN models to assess and predict collapse risks in tunnel engineering. Khakzad et al. [21] proposed a BN-based safety analysis method in process facilities.

Bayesian networks can be categorized into dynamic Bayesian networks and static Bayesian networks. In CCM, collapse accident data are usually scarce, and static Bayesian networks can still maintain high analytical performance under small sample conditions [44]. Since the collapse risk is mainly related to the current construction status, and the historical time series data are often difficult to obtain or lack of representativeness, the static Bayesian network is more suitable for the collapse risk analysis of the CCM. The challenges in obtaining accurate accident data complicates the determination of precise node failure probabilities in FTA or BN models. This issue is further exacerbated by the complex and diverse patterns and mechanisms of collapse, leading to inherent fuzziness and uncertainty in failure probabilities. As Gong et al. [22] noted, reasoning and predictions based on uncertain data inevitably result in uncertain outcomes, thereby complicating the provision of reliable decision-making guidance. To address these uncertainties, Hanss [45] emphasized that FST serves as an effective tool for managing uncertainty-related problems. Building on this, Abdolreza et al. [46] developed a risk assessment model based on FST specifically for evaluating risks in tunnel construction. Meanwhile, BN, as a probabilistic causal modeling technique, has been extensively applied in the metro industry for accident analysis and prediction. For instance, Yu et al. [44] utilized a BN model to investigate the causes of quality defects in construction projects. However, traditional BNs face limitations in probabilistic reasoning when exact probability values are difficult to ascertain.

To address these limitations, scholars have integrated FST with BN, proposing FBN to better handle the fuzziness and uncertainty inherent in collapse risk assessment. For instance, Zhang et al. [11] introduced an enhanced BN model by incorporating FST, specifically designed to evaluate the probability of collapse risk in metro projects. Subsequently, Zhang et al. [10] developed an FBN-based risk assessment model that incorporates expert credibility, further enhancing the reliability of collapse risk evaluation. Additionally, Zhang et al. [47] utilized FBN to analyze the risk of adjacent pipeline damage caused by collapse in tunnel engineering, providing theoretical support for ensuring tunnel construction safety. Similarly, Mamdouh et al. [48] employed FBN to monitor collapse risks in concrete pavement construction. In the context of sensitivity analysis for collapse accidents, Yu et al. [49] applied sensitivity analysis within the FBN framework to identify key factors contributing to collapse incidents, offering a scientific basis for collapse prevention. Furthermore, Yu et al. [50] utilized FBN to diagnose the causative factors of pipeline collapse failures, further expanding the application scope of FBN in collapse risk assessment. This integration of FST and BN not only addresses the limitations of traditional methods but also improves the accuracy and reliability of collapse risk assessment. Therefore, the FBN model can be utilized to study collapse accidents in CCM.

## 3. Research methodology and framework

FTA, BN and FST are widely used in risk analysis. These methods are jointly used to study collapse risk in this study. The basic theories of these methods and the research framework of this paper are presented in this section.

### 3.1. Fault tree analysis

FTA is based on a tree structure to decompose the causal factors of an accident and summarize the various combinations that lead to an accident [51]. In FTA, events are classified into top event, intermediate events, and bottom events. The relationships between events are represented using logical gates, with “OR” gates and “AND” gates being widely used [21]. FTA often employs Boolean algebraic expressions for qualitative descriptions and utilizes Boolean algebraic algorithms during the analysis process.

### 3.2. Bayesian networks

BN is also known as a belief network or directed acyclic graphical model. The BN mainly consists of conditional probability table (CPT) and directed acyclic graphical (DAG). The CPT is used to describe the conditional probability distribution of each node given different values of its parent nodes. The DAG is the structural foundation of a Bayesian network, consisting of nodes and directed edges to represent causal relationships between variables. A BN model can be represented by N=(X,T),P, where (X,T) denotes the DAG represented by the node. P refers to the CPT of each node, which represents the influence of the parent node on the child nodes [52]. In (X,T), $X=\left\{x_{1},x_{2},\cdots ,x_{n}\right\}$ is the set of all nodes in the DAG, each representing a variable [20]. T is the set of directed edges, which represent node probability dependencies. In BN, if a node has no parent node, the node is called the root node. If a node has no child nodes belonging to it, then this node is called a leaf node [52]. The directed edges describing the probabilistic dependencies of nodes are usually directed from the parent node to the child node. The relationship between $x_{i}$ and other nodes is conditionally independent when the parent node is determined, as shown in Eq (1).


$$P(x_{i}|y\left(x_{i}\right),B\left(x_{i}\right))=P(x_{i}|y\left(x_{i}\right))$$ (1)

Where the set of parent nodes $x_{i}$ is denoted as $y\left(x_{i}\right)$ and $B\left(x_{i}\right)$ is the set of all nodes except the parent nodes.

### 3.3. Fuzzy set theory

FST was first proposed by Zadeh [53] for decision analysis in uncertainty domains. A fuzzy number is a tool used in fuzzy theory to address problems that are difficult to define precisely or that have uncertainty. It describes the degree to which values belong to a set through the membership function. The commonly used fuzzy number processing methods are triangular, trapezoidal and Gaussian fuzzy numbers where the membership degree of each value reflects its belonging degree in the fuzzy set [54]. A triangular fuzzy number requires only three parameters and has a straightforward membership function, making it easy to operate [20]. Moreover, the highest membership degree of a triangular fuzzy number occurs at its most probable value, clearly expressing uncertainty. In contrast, the peak interval of a trapezoidal fuzzy number is wider, making it suitable for a smoother representation of fuzziness. The triangular fuzzy numbers are chosen in this study to transform the failure probabilities of the nodes into fuzzy numbers. The affiliation function relationship is shown in Eq (2). The fuzzy set is represented by $\tilde{M}$, where x is represented by an affiliation function $F_{\tilde{M}}\left(x\right)$ with interval [0,1]. The affiliation function $F_{\tilde{M}}\left(x\right)$ denotes the affiliation value of x in $\tilde{M}$.


$$F_{\tilde{M}}\left(x\right)=\left\{ \begin{array}{cc} 0 & x\leq g_{s}\\ \frac{x-g_{s}}{g_{m}-g_{s}} & g_{s}<x\leq g_{m}\\ \frac{g_{l}-x}{g_{l}-g_{m}} & g_{m}<x\leq g_{l}\\ 0 & x>g_{l} \end{array}\right.$$ (2)

Where $g_{m}$ is the center of the fuzzy subset, $g_{s}$ and $g_{l}$ are the upper and lower values.

### 3.4. Framework for collapse based on multi-state FBN

**Fig 1:**
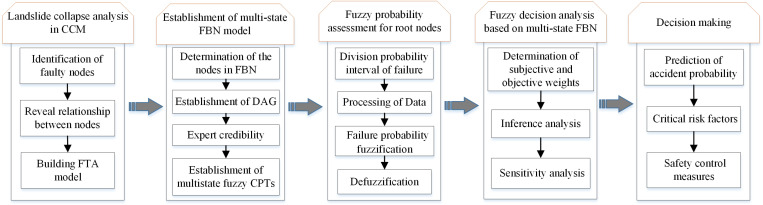
Evaluation process of collapse based on multi-state FBN.

The multi-state FBN model offers a viable approach for analyzing the failure of collapse. The process is illustrated in **Fig 1**, which consists of five steps: (1) Establishing FTA model: identifying risk factors based on historical data and constructing FTA model based on the mechanism of accident risk; (2) Constructing FBN model: establishing the failure network topology of FBN model based on FTA model and establishing CPT for non-root nodes; (3) Estimating the fuzzy failure probability of the root node: utilizing historical data and expert survey to determine the root node fuzzy failure probability; (4) Conducting decision analysis based on multi-state fuzzy Bayesian inference: including inference analysis and sensitivity analysis of the FBN model; (5) Risk decision: determine the collapse failure probability based on the inference results, and discover the check-points and the order of construction inspections during the construction process. Meanwhile, corresponding risk control measures are proposed.

## 4. Establishment of analytical modeling of collapse

### 4.1. Identification of faulty nodes

Numerous factors can lead to collapse incidents, with primary factors including integral sliding, reduced soil bearing capacity, and water inrush and sand inflow [15,55]. We collected 56 collapse cases constructed using the CCM method from 2006 to 2021. Based on chronological records in accident statistical reports, we analyzed the sequence of events leading to collapse. To systematically identify causal relationships among failure events, FTA was employed, integrating work breakdown structure and accident causation theory. Through this approach, we identified 9 intermediate factors and 16 bottom factors, tracing the progression from root causes to the final collapse. Since the bottom factors are probabilistically independent, their occurrence probabilities were treated as statistically independent variables in failure logic modeling. Additionally, these 16 bottom factors have been corroborated in other relevant studies, as shown in **Table 1**.

**Table 1:** Causal factors of collapse.

Intermediate event		Bottom event	Literature
Integral sliding Y_1_	Support instability Y_11_	Improper installation of horizontal supports X_1_	[10,56,57]
		Inadequate strength of support structures X_2_	[58–60]
	Improper excavation and support methods Y_12_	Improper arrangement of support systems X_3_	[60,61]
		Improper excavation methods X_4_	[10,62,63]
	Slope instability Y_13_	Overloading or excessive vibrations of surrounding soil X_5_	[58,64,65]
		Excessive slope steepness X_6_	[57,62]
Reduced soil bearing capacity Y_2_	Increased soil moisture content Y_21_	Long-term rainfall X_7_	[57,58,66]
		Adverse geological and hydrological conditions X_8_	[27,67,68]
	–	Soil erosion X_9_	[59,69,70]
Water inrush and sand inflow Y_3_	Pipeline damage Y_31_	Collapse above pipelines X_10_	[10,11,24]
		Pipeline leakage or damage X_11_	[61,71]
	Local seepage caused by improper drainage or excavation Y_32_	Excessive over-excavation or under-excavation X_12_	[66,71,72]
		Seepage in gravel layers at arch crowns X_13_	[58,59,69]
		Improper drainage methods X_14_	[73–75]
		Local formation water inrush and sand inflow X_15_	[10,11]
	–	Water inflow at the bottom of diaphragm walls X_16_	[67,63]

**Fig 2:**
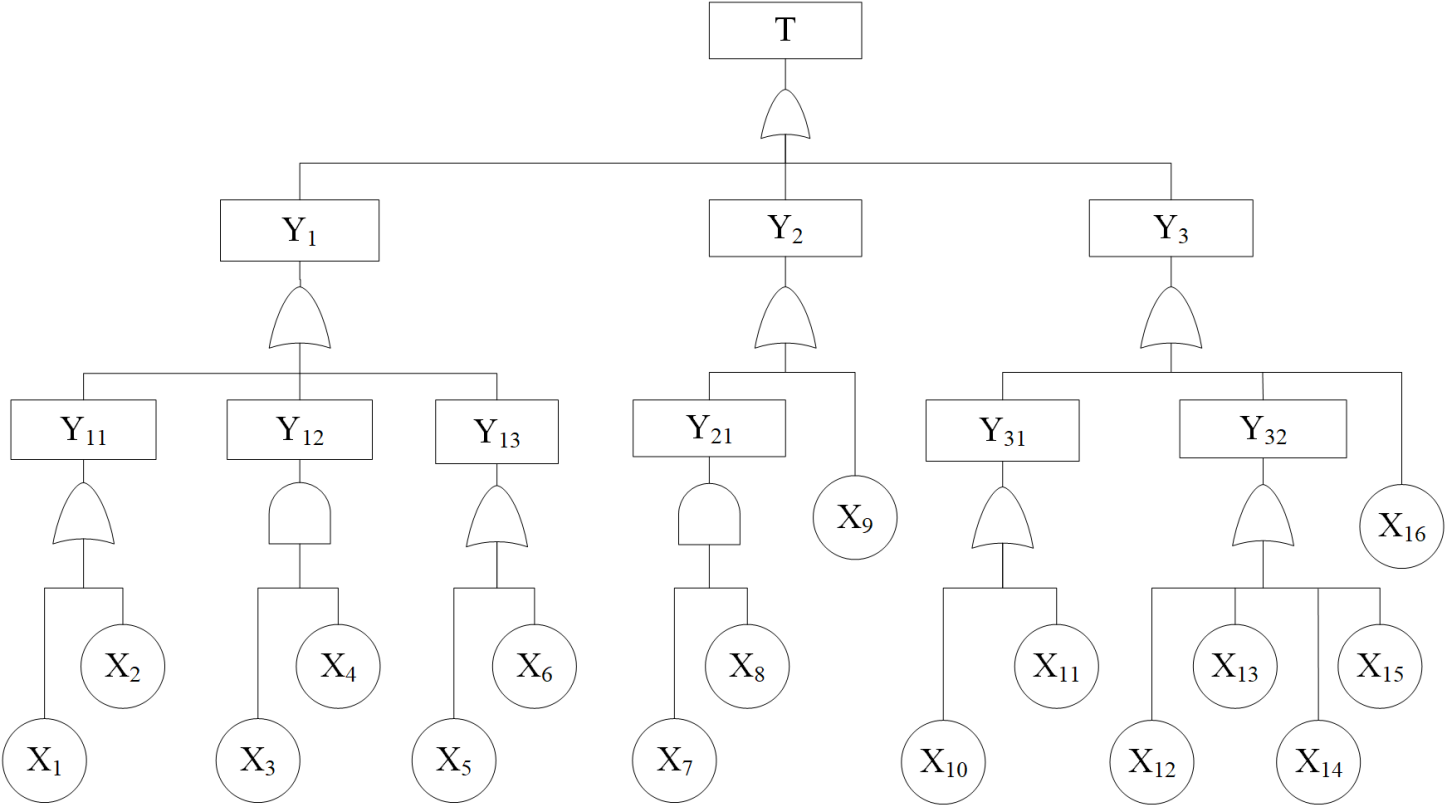
The FTA model of collapse.

Taking the collapse as the top event, the FTA model is constructed by considering the interrelationships between factors and utilizing logic gates, such as the “OR” and “AND” gates, as illustrated in **Fig 2**.

### 4.2. Establishment of multi-state FBN model

#### 4.2.1. Establishment of DAG.

**Fig 3:**
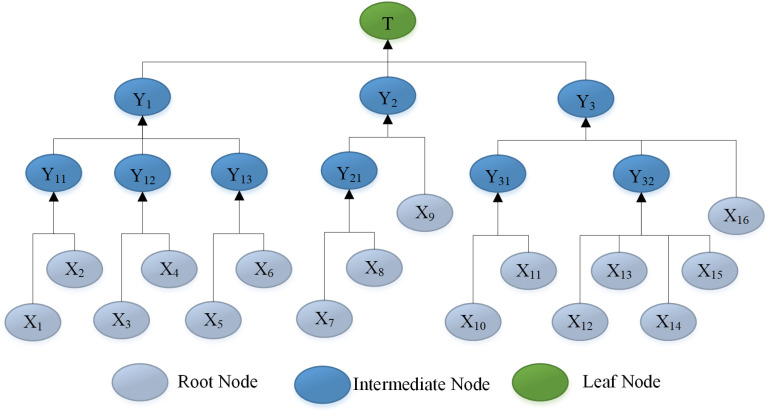
The BN model of collapse.

The structural configuration of the DAG is identical in FBN and FTA [21], and the DAG of the BN model can be established through the FTA transformation. The top event T (collapse), intermediate event ($Y_{1},\cdots Y_{3},Y_{11},\cdots Y_{32}$), and bottom event ($X_{1},X_{2},\cdots ,X_{16}$) in FTA are represented as the leaf node, intermediate node, and root node in the BN model, respectively. Subsequently, the corresponding nodes are connected through directed arcs to establish the DAG, as illustrated in **Fig 3**.

#### 4.2.2. Expert credibility.

Experienced experts can assist in determining the a priori probability of basic events when research data is insufficient [76]. However, it should be noted that expert judgment is influenced by factors such as work experience, job position and education, so the results may not always accurately reflect the experts’ actual knowledge [25]. Therefore, it is necessary to assign different weights to experts based on their professional levels. In practical applications, it is generally acknowledged that as individuals gain more work experience, hold higher job positions, and obtain higher education levels, their expertise and knowledge tend to increase [10,11]. Therefore, position, length of service, educational background, and age were selected as primary weighting criteria. Then they were further divided into four sub-criteria. The assignments of indicators from previous studies by Wang [77] and Zellers et al. [78] were referenced, and appropriate weights were assigned based on the hierarchical significance of each indicator in the rubric. A hierarchical table of expert judgment ability weights was formed, as shown in **Table 2**.

**Table 2:** The weight grading table of expert judgment ability.

Weight item	Weight $w_{i}$	Weighted Subsection	Sub-weight ${w_{i}}_{j}$	Weight item	Weight $w_{i}$	Weighted Subsection	Sub-weight ${w_{i}}_{j}$
Position	4	Project Manager	4	Educational background	2	Postgraduate and above	4
		Head of Department	3			Bachelor’s degree	3
		Project Manager	2			College Graduate	2
		Other managers	1			High School and below	1
Length of service	3	10 years and above	4	Age	1	50 years old and above	4
		6 ~9 years	3			40 ~ 49 years old	3
		2 ~ 5 years	2			30 ~ 39 years old	2
		Less than 2 years	1			Below 30 years old	1

The expert reliability is expressed as r, the higher value indicating the more reliable expert’s judgment. The $r_{m}$ of the expert can be calculated according to Eq (3). Where $w_{i}$ is the first level weight, and ${w_{i}}_{j}$ is the second level weight. Finally, the reliability of the experts is normalized by Eq (4). The normalized reliability of the experts is represented as $R_{m}$.


$$r_{m}=\sum {\mathrm{\backslash nolimits}}_{i=1}^{4}\sum {\mathrm{\backslash nolimits}}_{j=1}^{4}w_{i}{w_{i}}_{j}$$ (3)

$$R_{m}=\frac{r_{m}}{\sum_{i=1}^{4}w_{i}w_{ij}}$$ (4)

#### 4.2.3. Establishment of multi-state CPTs.

During the CCM construction process, localized and manageable small-scale collapses can be acceptable under certain conditions [33]. Such collapses do not have a substantive impact on construction progress, personnel safety, or property and typically do not require additional measures to address them [32]. However, some collapse incidents may lead to work stoppages at the construction site, casualties, or significant economic losses, necessitating responsive measures to control risks and minimize damages [6]. Therefore, based on whether measures need to be taken to respond after a collapse incident, the failure modes of collapse incidents can be categorized into no-failure, moderate-failure, and severe-failure for the intermediate nodes and leaf nodes. These states can be represented by 0, 0.5 and 1 respectively. The no-failure state refers to the absence of failure events or minor failures that do not affect construction and normal operation. The moderate-failure state means that measures need to be taken to mitigate the occurrence of the failures, accompanied by some economic losses. The severe-failure state may result in site shutdown, personnel injuries, and significant economic losses. Each expert needs to assess the possible failure states of node T under different combinations of $y_{1}$, $y_{2}$, and $y_{3}$. Here, the $y_{1}$, $y_{2}$, and $y_{3}$ represent the various failure states of $Y_{1}$, $Y_{2}$, and $Y_{3}$, respectively. For example, $P(T=1|y_{1}=0,y_{2}=0,y_{3}=0)$ represents the probability of severe-failure of node T when nodes $Y_{1}$, $Y_{2}$, and $Y_{3}$ have a failure state of 0. The expert’s judgment results need to be combined with their reliability for comprehensive assessment. Assuming a certain expert’s judgment for $P(T=1|y_{1}=0,y_{2}=0,y_{3}=0)$ is 0.08, and their reliability is calculated as 0.7 using Eq (4). Considering the expert credibility, the adjusted result should be 0.056. Subsequently, the calculated results are normalized to obtain the CPT of the non-root node.

### 4.3. Fuzzy probability assessment for root nodes

#### 4.3.1. Division probability interval of failure.

During data surveys, it is challenging for experts to accurately estimate the probabilities of nodes. The division of probability intervals allows the experts to provide a range of probabilities rather than a definite value. The width of the interval reflects the reliability of the assessment. Dawes [79] has highlighted that dividing the intervals into 5–9 levels ensures higher accuracy of the data. A few classifications do not meet the accuracy requirements, while more classifications hinder the collection of expert opinions. To address the difficulties posed by excessive categories in expert evaluations, the seven semantic variables were simplified based on the 9-level semantic variables proposed in *Engineering Psychology and Human Performance* [80] and Zhang et al. [11]. These semantic variables will be used to describe the probability intervals for the occurrence of root nodes. The division of probability intervals and the respective triangular fuzzy numbers are shown in **Table 3**.

**Table 3:** The possibility intervals of root nodes and triangular fuzzy probability numbers.

Possibility interval(k)	Linguistic term	Triangular fuzzy probability numbers		
Lower boundary (g_s_)	Mean (g_m_)	Upper boundary (g_l_)
1	Very low possibility	0	0.025	0.05
2	Low possibility	0.05	0.125	0.2
3	Nearly low possibility	0.2	0.275	0.35
4	Moderate possibility	0.35	0.425	0.5
5	Nearly hight possibility	0.5	0,725	0.75
6	High possibility	0.75	0.825	0.9
7	Very high possibility	0.9	0.95	1

Assuming that n experts participate in the assessment. It is assumed that the expert assesses the event $X_{i}$ falling in the interval k denoted as $P_{km}$. The fuzzy possibility interval of $X_{i}$ is denoted by $P_{X_{i}}$. As shown in Eq (5), the assessment results of the experts are multiplied by their expert credibility and then summed to obtain $P_{X_{i}}$.


$$P_{X_{i}}=\sum {\mathrm{\backslash nolimits}}_{m=1}^{n}\left(\frac{R_{m}}{\sum_{m=1}^{n}R_{m}}\times P_{km}\right)≅\left(g_{s}\;\;\prime \;\;\text{ ,}\text{g}m\;\;\prime \;\;\text{ ,}\text{g}l\prime \right)$$ (5)

Where $\frac{R_{m}}{\sum_{m=1}^{n}R_{m}}$ represents the reliability of the assessment data provided by the m -th expert, and $R_{m}$ is calculated by Eq (4).

#### 4.3.2. Processing of data.

Suppose an expert determines the probability of an event occurring in the interval k. When the expert confidence is less than 1, there is bound to be a probability that $\text{1-}R_{m}$ falls in the other 6 intervals, which was called the residual probability. The residual probability was often neglected in previous studies, leading to some potential information being ignored. According to the Gaussian distribution law, it is known that the probability of occurrence of an event decrease as it gets farther from the expected value [10,11]. Therefore, the formula for the distribution of the residual probability $\text{1-}R_{m}$ is provided, as is shown in Eq (6).


$$P_{{x_{m}}^{i}}=\left\{ \begin{array}{cc} \frac{\left(a_{k}-a_{k-i}\right)}{\sum_{j=1}^{k-1}\left(a_{k}-a_{j}\right)}\times \frac{1-R_{m}}{2} & 1\leq i\leq k-1\\ R_{m} & i=k\\ \frac{\left(a_{8+k-i}-a_{k}\right)}{\sum_{j=k+1}^{7}\left(a_{j}-a_{k}\right)}\times \frac{1-R_{m}}{2} & k+1\leq i\leq 7 \end{array}\right.$$ (6)

Where $R_{m}$ is obtained by Eq (4), $a_{k}$ represents the lower bound of the k -th fuzzy interval, and i(1≤i≤7) denotes the interval where the residual probability is located.

#### 4.3.3. Calculation of failure probability.

It is assumed that n experts and engineering managers participate in the questionnaire. The average failure probability of root node $X_{i}$ is calculated by using arithmetic average, as shown in Eq (7). According to the “3σ criterion”, it is known that the random variables are accurate up to 99.7% within the interval [E(P)−3σ,E(P)+3σ] [11,81]. E(P) represents the expectation, and σ represents the standard deviation. Therefore, the “3σ criterion” is applied to calculate the eigenvalues of the triangular fuzzy numbers, and the relevant formulas are shown in Eqs. (8) to (10).


$$\overset{―}{P_{{x_{i}}^{m}}}=\frac{\sum_{m=1}^{n}P_{{x_{i}}^{m}}}{n}$$ (7)

$$u=E\left(P\right)=\sum {\mathrm{\backslash nolimits}}_{k=1}^{7}\left(g_{k}\times P_{{x_{i}}^{m}}\right)$$ (8)

$$\sigma =\sqrt{D\left(P\right)}=\sqrt{\sum {\mathrm{\backslash nolimits}}_{k=1}^{7}\left[{\left(g_{k}-E\left(P\right)\right)}^{2}\times P_{{x_{i}}^{m}}\right]}$$ (9)

a=μ−3σ;b=μ+3σ (10)

Where $g_{k}$ represents the centroid value of the triangular fuzzy function within the k -th interval in Table 2. a, b, and μ denote the characteristic values of the triangular fuzzy number.

After the above process, the root node is still a fuzzy value. The fuzzy numbers need to be converted into a definite number to represent this set of fuzzy numbers, and this process is known as defuzzification. The most common defuzzification methods include α-estimation, equipartition [82] and center-of-mass [83]. In the research of Zhang [10] and Zhang [11] et al. it is noted that the α-estimation method can effectively reduce the loss of information in the defuzzification process. Therefore, the α-estimation method is used to complete the conversion process as in Eq (11).


$$Val\left(P_{X_{i}}\right)=\frac{\int_{0}^{1}Average\left(F_{\alpha }\right)\times f\left(\alpha \right)d\alpha }{\int_{0}^{1}f\left(\alpha \right)d\alpha }$$ (11)

$$Average\left(F_{\alpha }\right)=\frac{u_{\alpha }+v_{\alpha }}{2}$$ (12)

Where $Val\left(P_{X_{i}}\right)$ is the exact value after defuzzification and $F_{\alpha }=\left\{x\left|F\left(x\right)\right\rangle \alpha \right\}$ is the α-level set of F. $Average\left(F_{\alpha }\right)$ is the mean value of the α-level set, whose value is calculated by Eq (12).

**Fig 4:**
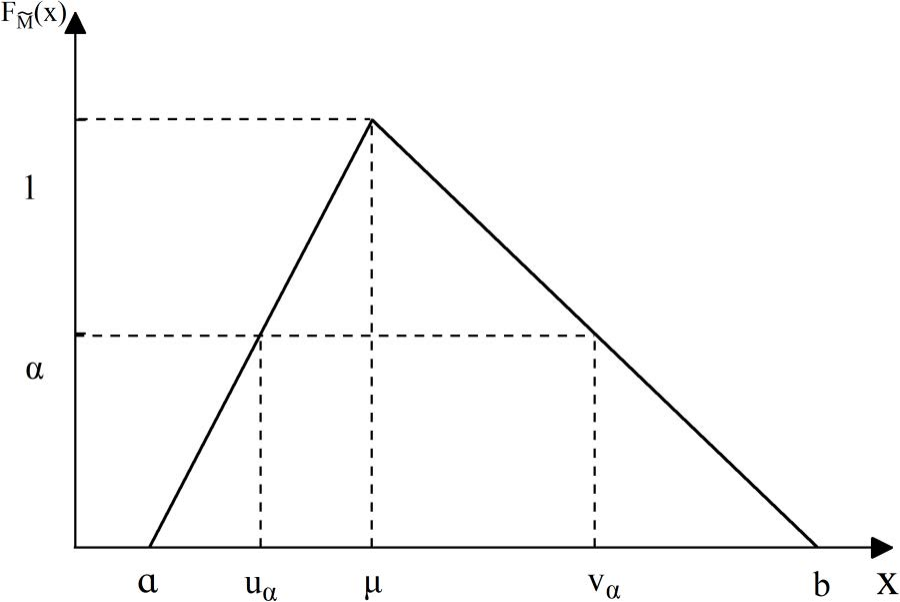
Membership function of a triangular fuzzy number $P_{X_{i}}$.


Where $u_{\alpha }$ and $v_{\alpha }$ are the upper and lower bounds of the α-level set, as shown in **Fig 4**. $u_{\alpha }$ is calculated by Eq (13) and $v_{\alpha }$ is calculated by Eq (14). In general, α=0.5, f(α)=1 [76]. Accordingly, $Val\left(P_{X_{i}}\right)$ also can be calculated by Eq (15). The prior probability of the root node in BN can be obtained according to the above calculation procedure.

$$u_{\alpha }=\left(\mu -a\right)\times \alpha +a$$ (13)

$$v_{\alpha }=b-\left(b-\mu \right)\times \alpha$$ (14)

$$Val\left(P_{X_{i}}\right)=\frac{\frac{1}{2}\int_{0}^{1}\left(\mu -a\right)\times \alpha +a+b+\left(b-\mu \right)\times \alpha d\alpha }{\int_{0}^{1}d\alpha }=\frac{a+2\mu +b}{4}$$ (15)

#### 4.3.4. Correction of failure probability.

While expert knowledge is an important source for obtaining risk assessment data, relying solely on expert knowledge to obtain the failure probability of the root node is not accurate. To address this issue, a combination of objective weight (project historical data) and subjective weight (expert survey data) was used to modify the fuzzy failure probability of the root node, as shown in Eq (16). In the equation, $Val{\left(P_{X_{i}}\right)}^{\prime }$ is the corrected root node failure probability, $k_{1}$ denotes the objective weight, and $k_{2}$ represents the subjective weight. The values of $k_{1}$ and $k_{2}$ are obtained by the experts according to the actual situation in the field.


$$Val{\left(P_{X_{i}}\right)}^{\prime }=k_{1}\times Val\left(P_{A_{i}}\right)+k_{2}\times P_{i}$$ (16)

In this study, 56 cases of collapse associated with the CCM construction method were collected, covering the period from 2006 to 2021. These cases were considered as project history data. The occurrence frequency of the root node in the sample cases was analyzed, and the node relative frequency was used as a substitute for the probability of node occurrence. The detailed results are presented in **Table 4**.

**Table 4:** Statistics of causal factors of collapse.

Root node	Frequency/Times	Relative frequency (P_i_)	Root node	Frequency/Times	Relative frequency (P_i_)
X_1_	9	0.080	X_9_	5	0.045
X_2_	8	0.071	X_10_	3	0.027
X_3_	16	0.143	X_11_	7	0.063
X_4_	11	0.098	X_12_	9	0.080
X_5_	7	0.063	X_13_	3	0.027
X_6_	1	0.009	X_14_	10	0.089
X_7_	9	0.080	X_15_	3	0.027
X_8_	8	0.071	X_16_	3	0.027

## 5. Fuzzy decision analysis based on multi-state FBN

After obtaining the failure probability of the root node and the conditional probability of the non-root nodes, the established multi-state FBN was utilized for decision analysis. Ren [84] and Zhang [25] have demonstrated that FBN can predict the probability distribution of potential safety risks and identify the underlying risk factors. This paper primarily focuses on inference analysis and sensitivity analysis of collapse using a multi-state FBN.

### 5.1. Inference analysis

The inferential analysis aims to deduce the probability distribution of the leaf nodes T considering the combination of root nodes and intermediate nodes [25]. The probability distribution of the root node is represented by P(T=t), which is calculated by Eq (17). The root node prior probability is input as the initial state of the system during inference. P(T=t) can be used to assess the potential risk level of T and assist decision-makers in implementing reasonable preventive or control measures. A cut set is a set of basic events that can trigger a system failure. When any of the fundamental events in the cut set is removed, the system failure does not occur, and such a cut set is called the minimum cut set. There is no need to determine the minimum cut set in FBN, making FBN significantly more efficient than TF in terms of computational and inferential analysis.


$$\begin{array}{cc} & P\left(T=t\right)≅\sum {\mathrm{\backslash nolimits}}_{1}^{Q_{1},Q_{2},\cdots ,Q_{n},R_{1},R_{2},\cdots ,R_{m}}[P(T=t|X_{1}=x_{1},X_{2}=x_{2},\cdots ,X_{n}=x_{n},Y_{1}=y_{1},Y_{2}=y_{2},\cdots Y_{m}=y_{m})⊗\\ & \;\;\;\;\;\;\;\;\;\;\;\;\;\;\;\;\;\;P\left(X_{1}=x_{1},X_{2}=x_{2},\cdots ,X_{n}=x_{n},Y_{1}=y_{1},Y_{2}=y_{2},\cdots Y_{m}=y_{m}\right)]\\ & t=\left\{t_{1},t_{2},\cdots ,t_{P}\right\};x_{i}=\left\{{x_{i}}^{1},{x_{i}}^{2},\cdots {x_{i}}^{Q_{i}}\right\};y_{j}=\left\{{y_{j}}^{1},{y_{j}}^{2},\cdots {y_{j}}^{R_{j}}\right\};i=1,2,\cdots n,j=1,2,\cdots m; \end{array}$$ (17)

Where $\left\{t_{1},t_{2},\cdots ,t_{P}\right\}$ is the range of P states of the leaf node T, $\left\{{x_{i}}^{1},{x_{i}}^{2},\cdots {x_{i}}^{Q_{i}}\right\}$ is the range of $Q_{i}$ states of the root node $X_{i}$, and $\left\{{y_{j}}^{1},{y_{j}}^{2},\cdots {y_{j}}^{R_{i}}\right\}$ is the range of $R_{j}$ states of the intermediate node $Y_{j}$. $P(T=t|X_{1}=x_{1},X_{2}=x_{2},\cdots ,X_{n}=x_{n},Y_{1}=y_{1},Y_{2}=y_{2},\cdots Y_{m}=y_{m})$ denotes the conditional probability distribution of the leaf node T, and $P\left(X_{1}=x_{1},X_{2}=x_{2},\cdots ,X_{n}=x_{n},Y_{1}=y_{1},Y_{2}=y_{2},\cdots Y_{m}=y_{m}\right)$ denotes the joint probability distribution of root and intermediate nodes.

### 5.2. Sensitivity analysis

In engineering practice, decision-makers make efforts to identify the key factors contributing to collapse and the order of risk inspections [73]. The determination of key inspection points and the order of risk inspections are mostly determined by experts, and there could be a certain deviation in judgment results. Such treatment may miss the best time to control the risk and cause serious damage. Sensitivity analysis aims to infer the potential consequences associated with specific factors and achieve real-time fault diagnosis through analysis of sensitivity indicators. By utilizing sensitivity indicators, critical check-points in the construction process can be accurately identified. In this study, sensitivity analysis was conducted using the Sensitivity Performance Measure (SPM) proposed by Zhang et al. [11], as depicted in Eq (18).


$$SPM\left(X_{i}\right)=\frac{1}{Q_{i}}\sum {\mathrm{\backslash nolimits}}_{i}^{Q_{i}}\left|\frac{P(T=t|X_{i}=x_{i})-P\left(x=t\right))}{P\left(T=t\right)}\right|$$ (18)

Where t denotes the failure state of the leaf node T, and $x_{i}$ is the failure state of the root node $X_{i}$. The $SPM\left(X_{i}\right)$ of $X_{i}$ is greater, the influence of $X_{i}$ is greater on T. When the value of $SPM\left(X_{i}\right)$ is 1, $X_{i}$ has a high probability of being the direct cause of the accident. In engineering practice, if the state of $X_{i}$ is set to $q_{i}$ ($X_{i}={x_{i}}^{q_{i}}$), $SPM\left(X_{i}\right)$ can be calculated by Eq (19). The sensitivity analysis of the root node can realize real-time risk monitoring.


$$SPM\left(X_{i}\right)≅\frac{1}{Q_{i}-1}\sum {\mathrm{\backslash nolimits}}_{1}^{1,2,\cdots q_{i}-1,q_{i}+1,\cdots Q_{i}}\left|\frac{P(T=t|X_{i}=x_{i})-P(T=t|X_{i}={x_{i}}^{q_{i}}))}{P(T=t|X_{i}={x_{i}}^{q_{i}})}\right|$$ (19)

### 5.3. Decision making

Inference analysis enables the estimation of the occurrence probability of collapse, aiding decision-makers in adjusting and optimizing construction plans to mitigate risks within manageable limits. Sensitivity analysis can identify the key risk-causing factors and the order of risk inspection in collapse, so that decision-makers can devote more energy to the control of key risk-causing factors. Moreover, real-time fault diagnosis based on sensitivity ranking enables precise control of risk management by facilitating timely intervention and remedial actions.

## 6. Case study

### 6.1. Engineering situations

A metro station is selected as a case study to analyze the risk of collapse. The station is being constructed using the CCM construction method, and the surrounding environment of the station is complex. At the bottom of the pit, there is a layer of silt ranging from 6.5 to 15 meters, which exhibits high permeability. The building envelope adopts underground diaphragm wall, and the support system in the pit adopts caisson pile and inner support of steel pipe. The excavation of the foundation pit may lead to slip accidents along the level.

### 6.2. Data gathering and fuzzy failure probability estimation

#### 6.2.1. Determination of root node prior probability.

According to the section 4.3.1 and 4.3.2, questionnaires were designed to determine the root node prior probability. Questionnaires were distributed through email consultation and on-site research for experienced experts. The purpose and content of the questionnaire have been communicated in writing to the participants, and all individuals participating in the survey provided their written consent. A total of 100 questionnaires were distributed, and 89 valid questionnaires were returned. The background information of the questionnaire respondents is presented in **Table 5**. Among respondents, 57.3% were experts with more than 5 years of working experience, while 51.7% were experts with a bachelor’s degree or above. The respondents evaluated the probability interval for the occurrence of the root node based on **Table 3**.

**Table 5:** Background of the questionnaire participants.

Item	Content	Frequency	Percentage
Length of service	5 years and above	51	57.3%
Educational background	Bachelor’s degree or above	46	51.7%
Working company	Construction Company	39	43.8%
	Design company	14	15.7%
	Supervisory and consulting company	7	7.9%
	University scholar	12	13.5%
	Owner	17	19.1%

**Fig 5:**
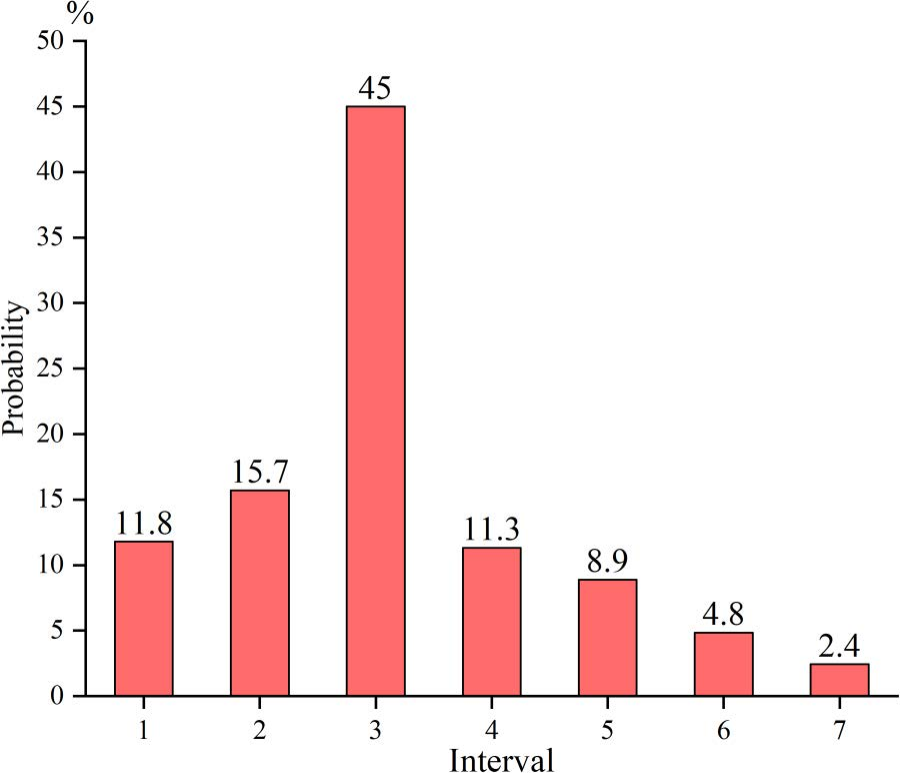
The interval distribution of residual probability.

Based on the background information of the interviewees, the expert credibility $R_{m}$ was calculated by Eqs. (3) and (4). In the case of unreasonable root node $X_{\text{14}}$ (Improper drainage methods), the basic information of expert m was as follows: occupation - other manager, work experience - two and a half years, education - bachelor’s degree, and age - 33 years old. This expert believes that the maximum probability of failure of the node $X_{\text{14}}$ falls in the 3rd interval, namely interval [0.2,0.35] (**Table 3**). Then the initial importance of this respondent can be calculated by $r_{1}=4\times 1+3\times 2+2\times 3+2\times \text{1=18}$, whose normalized expert credibility is 0.45. The residual probability is 0.55, distributed over the remaining 6 probability intervals. By Eq (6), the average probability distribution of the remaining intervals can be determined, as illustrated in **Fig 5**. The probability distributions of the remaining expert interview results were obtained according to the above process.

After obtaining the probability distribution from expert interviews, the average failure probability of each root node was calculated by Eq (7). Subsequently, the average failure probabilities were transformed into fuzzy probability evaluation results using Eqs (8) to (10). The fuzzy probability evaluation results for the root nodes in the FBN model are presented in **Table 6**.

**Table 6:** Root node fuzzy failure probability evaluation results.

Root node	Fuzzy probability assessment			Root node	Fuzzy probability assessment		
a	μ	b	a	μ	*b*	
X_1_	0.043	0.049	0.054	X_9_	0.076	0.080	0.083
X_2_	0.054	0.058	0.061	X_10_	0.023	0.026	0.028
X_3_	0.202	0.204	0.206	X_11_	0.100	0.120	0.140
X_4_	0.041	0.044	0.046	X_12_	0.069	0.070	0.071
X_5_	0.054	0.055	0.056	X_13_	0.034	0.037	0.040
X_6_	0.015	0.017	0.019	X_14_	0.159	0.161	0.063
X_7_	0.134	0.136	0.138	X_15_	0.017	0.021	0.024
X_8_	0.040	0.042	0.044	X_16_	0.043	0.044	0.045

#### 6.2.3. Determination of multi-state CPT for non-root nodes.

The CPT of non-root nodes is typically composed of conditional probabilities. Each expert is required to indicate the probability of a child node experiencing failure in different combinations of parent node states. Due to space limitations, only the CPTs of leaf nodes are presented, as shown in **Table 7**.

**Table 7:** Multi-state CPT of leaf node T.

$y_{1}$	$y_{2}$	$y_{3}$	$P(T=t|y_{1},y_{2},y_{3}),t=0,0.5,1$		
			t=0	t=0.5	t=1
0	0	0	0.837	0.125	0.038
0	0	0.5	0.783	0.158	0.059
0	0	1	0.773	0.166	0.061
0	0.5	0	0.738	0.178	0.084
0	1	0	0.708	0.185	0.107
…	…	…	…	…	…
1	0	1	0.105	0.225	0.670
1	0.5	1	0.080	0.198	0.722
1	1	0	0.064	0.202	0.734
1	1	0.5	0.051	0.207	0.742
1	1	1	0.035	0.189	0.776

### 6.3. Fuzzy decision analysis for collapse

#### 6.3.1. Probabilistic reasoning.

**Fig 6:**
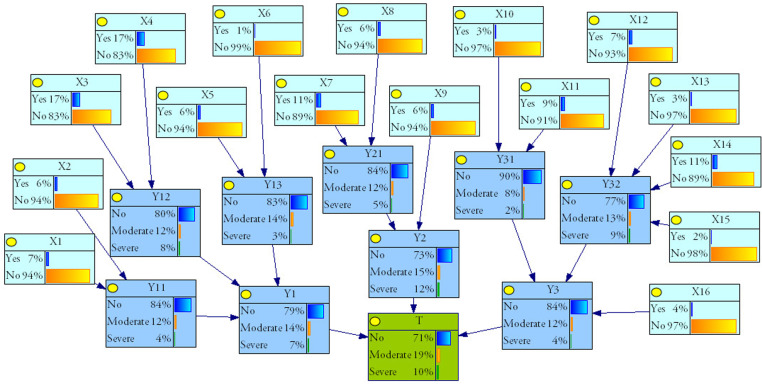
Failure probability prediction results of collapse.

During the inference analysis, it is necessary to denitrify the fuzzy evaluation results from Table 6. For example, the fuzzy evaluation result of the root node $X_{1}$ is (0.043,0.049,0.054). By applying Eq (15) for defuzzification, we obtain $Val\left(X_{1}\right)$ as 0.049. Through interviews with seven experts, including three university experts and four site managers, we determine the values of both the objective weight $k_{1}$ and the subjective weight $k_{2}$ in the project. Subsequently, using Eq (16) and the accident statistics data from Table 4, we correct the root node failure probability. As a result, the corrected failure probability $Val{\left(P_{X_{i}}\right)}^{\prime }$ is 0.065. Utilizing the corrected as the initial data input in the Genie software for inference, the results are shown in **Fig 6**. The above results indicate that the probability of severe-failure state is 10%, the probability of moderate-failure state is 19%, and the probability of no-failure state is 71%, which is consistent with the actual situation.

#### 6.3.2. Sensitivity analysis and risk management.

**Fig 7:**
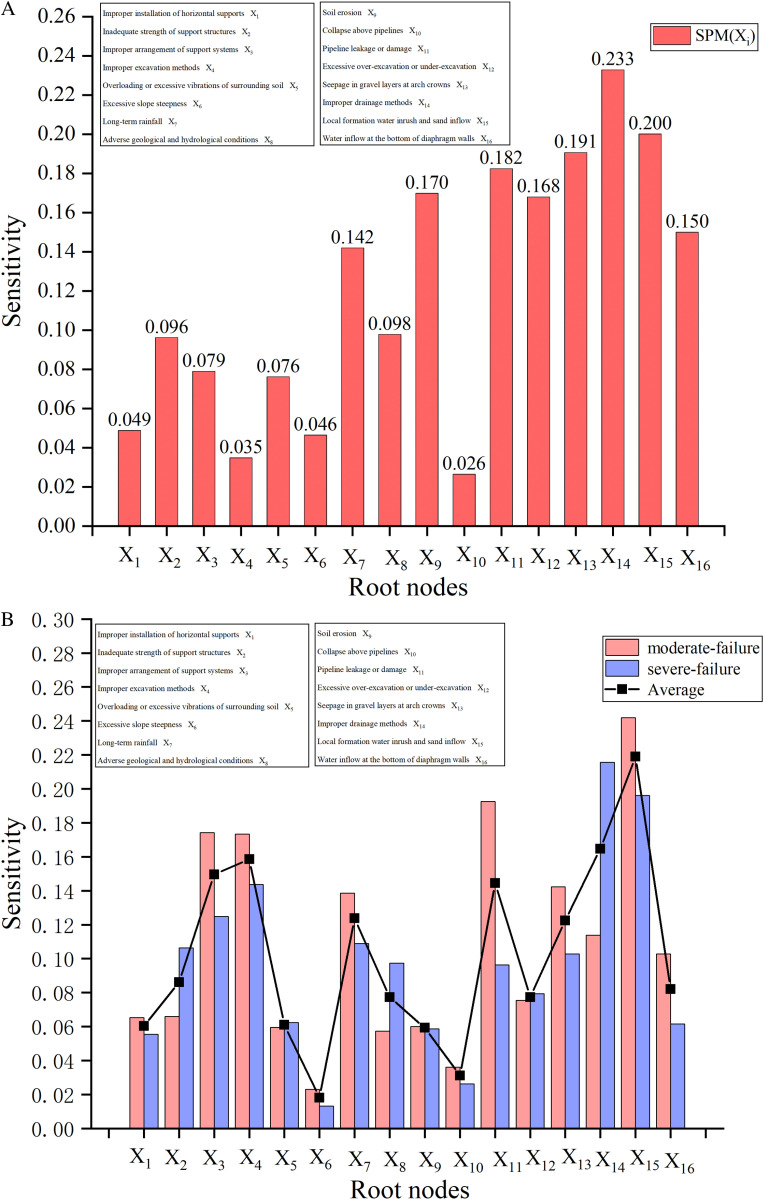
Identification of sensitive factors for collapse. (a) Scenario A (prior probabilities) (b) Scenario B (actual state of root nodes).

In scenario A (a priori probability), the actual values of the root nodes are unknown. The sensitivity indicator $SPM\left(X_{i}\right)$ of each root node is calculated by Eq 18, as depicted in **Fig 7(a)**. The $X_{\text{14}}$, $X_{\text{15}}$, $X_{\text{13}}$ and $X_{11}$ are the most likely causative factors for collapse. Therefore, waterproofing and drainage measures should be strengthened in time during the construction process. The supervision and inspection of pipelines should be enhanced to prevent the collapse caused by the increase of water content in the soil around the pit. The remaining factors are ranked in order of sensitivity is $X_{12}>X_{16}>X_{7}>X_{8}>X_{2}>X_{3}>X_{5}>X_{1}>X_{6}>X_{10}$.

In scenario B (actual state of the root nodes), the actual failure state of root nodes is determined through field monitoring. The $SPM\left(X_{i}\right)$ of each root node is calculated by Eq (19), as shown in **Fig 7(b)**. When a medium-scale collapse occurs, the direct causes of failure are identified as $X_{15}$, $X_{11}$, $X_{3}$ and $X_{4}$. To prevent moderate-scale collapse, it is crucial to strengthen inspections of site pipelines and support structures. In the case of a severe-scale collapse, factors $X_{14}$, $X_{15}$, $X_{4}$ and $X_{7}$ have a greater impact on the accident. Improper drainage methods directly increase soil moisture content, rapidly reduce soil stability, and can promptly trigger collapses, while the effects of long-term rainfall are cumulative and harder to control. Consequently, in collapse incidents, problems associated with drainage measures are considered more critical and immediate compared to long-term rainfall. Preventive measures for severe-scale collapse should involve implementing drainage and waterproofing measures, and adopting appropriate excavation and support methods. It is worth noting that both factors $X_{4}$ and $X_{15}$ are important in controlling moderate-scale and severe-scale collapse, and should receive special attention.

In different contexts, there may be some overlap among key risk factors, but their sensitivity levels may vary. This method can be used to determine the sensitivity ranking of each risk factor, providing a convenient way for decision-makers to identify the key check-points and the order of risk inspections during the construction process. Emphasizing these critical check-points during the construction process can effectively reduce the occurrence of collapse.

## 7. Discussion and implications

Many studies have introduced accident assessment and prediction for collapse accidents**.** However, the management of collapse risks in the CCM has not received widespread attention. Particularly, some safety risks are inherent in construction methods. This study is dedicated to finding the inherent factors that cause collapse accidents from the construction process of CCM. In previous studies, the collapse risk in metro construction mainly focuses on tunnel collapse accidents [10,11,19]. In this study, 56 collapse accidents using CCM were collected to excavate potential risk causes from the construction process. By analyzing the accident cases and previous research results, this study has identified 9 intermediate factors and 16 bottom factors contributing to collapse, and built FTA and FBN models accordingly. The method can be used to analyze the probability of collapse, identify key check-points, and establish the order of risk inspection during the construction process. This study fills the gap in research on collapse from the CCM construction process. The results can be used for risk control of collapse in CCM construction methods and provide recommendations for decision-making.

In terms of analytical methods, this study proposed a multi-state FBN, which was constructed based on the relationships among these factors in the FTA. Additionally, to enhance the descriptive capability of BN models in representing actual failure probabilities, this study introduced multi-state of node. And fuzzy subsets were used to describe the failure probability of the nodes, which reduced the difficulty of obtaining the exact probability of the node. This differs with previous researchers, whose failure probability of traditional BN model nodes was often expressed using deterministic probabilities. For instance, Sousa [15] and Pei [61] employed the BN model to study the accident probability of tunnel collapse safety risk during metro construction. This study integrated FST and BN to enhance the ability of the model to deal with fuzzy and uncertainty problems, making it more practical for engineering applications.

Furthermore, this study classified the risk of collapse into small, moderate, and severe scales for refined risk management. In previous studies, accidents were typically defined as either “occurrence” or “non-occurrence”. Zhang et al. [11] utilized FBN to analyze and predict the probability of pipeline collapse accidents, yet they did not differentiate between the scales of collapse accidents. As different scales of accidents require distinct approaches for response and handling, this study handled this problem by using the three scales to quantify the risk of collapse. The division of multi-states allows for distinguishing different failure states, enabling separate predictions for each specific failure state. In case study, the occurrence probability of three failure states was analyzed. The results demonstrated that the probabilities of no-failure, moderate-failure, and severe-failure were 71%, 19%, and 10%, respectively. The sensitivity analysis of the metro station showed that excessive over-excavation or under-excavation and water inflow at the bottom of diaphragm walls had the greatest impact on collapse. Subsequently, factors such as long-term rainfall, adverse geological and hydrological conditions, inadequate strength of support structures, and improper arrangement of support systems contributed to the collapse. The results are different from tunnel collapses. In previous study, Zhang et al. [25] analyzed the factors involved in tunnel collapse accidents, and identified the top 5 critical factors in the tunnel collapse process: soil cohesion, horizontal relative distance, vertical relative distance, compressive modulus, and friction angle. The professional monitoring, construction techniques, and quality management mentioned by Zhang et al. [25] are all significant factors leading to both types of collapse accidents.

In practice, key check-points are more important to ensure safety, and decision-makers are more inclined to invest more money and effort in them. Sensitivity analysis of BN serves as a vital tool to determine the order of critical factors. Zhang [25] and Sun [19] used sensitivity indicators of the BN model to identify the key factors causing tunnel collapse accidents. Notably, this study further conducted sensitivity analyses especially for moderate and severe collapse. It was observed that there may be overlapping factors among different levels of collapse, but the importance of key risk-causing factors varies. In the case of medium-scale collapse, factors such as Local formation water inrush and sand inflow, Pipeline leakage or damage, Improper excavation methods, Improper excavation methods have a greater impact on the accident. To prevent medium-scale collapse, it is crucial to strengthen inspections of site pipelines and support structures. This differs from the risk of tunnel collapse previously studied by Zhang et al. [10]. In the study of Zhang et al. [10], it is suggested that the five key factors for medium-scale tunnel collapse are Continuity of management, Quality of construction, Excavation method, Continuity of support and proper supervision. In the case of medium-scale collapse incidents in tunneling accidents, these incidents are often associated with management factors. And factors Improper drainage methods, Local formation water inrush and sand inflow, Improper excavation methods, Long-term rainfall have a greater contribution on the severe-scale collapse. Preventive measures for large-scale collapse should involve implementing drainage and waterproofing measures, and adopting appropriate excavation and support methods. While in the study of Sun [19], it was concluded that large-scale tunnel collapses are mostly related to material qualification, support timeliness, geology mutation and construction normalization. It is worth noting that both excavation methods, water inrush and sand inflow are important in controlling medium-scale and large-scale collapse, and should receive special attention. Therefore, the collapse of different scales should be targeted to control the key factors to improve the efficiency of risk management.

This study solves the research gap concerning the analysis and prediction of collapse from the perspective of CCM, and further enhances the previous research results. From a theoretical standpoint, it provides an analytical framework for collapse in CCM and offers methods for predicting different failure states and analyzing key factors. In terms of practical application, the research results can be used to identify key check-points in the construction process and predict the collapse risks.

## 8. Conclusion

The frequent occurrence of collapse has resulted in serious losses in metro construction projects. However, due to differences in construction processes, the risk associated with collapse varies among construction methods. Few studies have analyzed and predicted the risk of collapse from the perspective of construction methods. This study aims to establish a multi-state FBN model to investigate the collapse during the CCM construction process, providing a reference for reducing metro construction risks.

Firstly, the influencing factors of collapse are determined based on the engineering accident data to build FTA and BN model. Subsequently, the FST is used to represent the fuzziness of node probability when describing the failure probability of root nodes, and the uncertainty of the connection between nodes was described using CPT. The problem of obtaining failure probabilities for root nodes and dealing with the uncertainty of node connections between nodes in collapse was better solved. It should be noted that we combined expert interviews with accident statistics to obtain the prior probabilities and CPTs, improving the reliability of data. Furthermore, the credibility level and residual probability of objective expert judgment were considered. The method’s validity was demonstrated through a metro station project which is constructed by CCM. By predicting the probability of collapse and identifying sensitive factors, decision-makers can make informed judgments and effectively reduce the probability of collapse in CCM by controlling key factors.

However, this paper also has some limitations. Firstly, the established multi-state FBN model does not include all the factors contributing to collapse, and some relatively uncertain factors need to be further determined. Secondly, the collection of collapse cases did not include some unreported collapses. Future studies can focus on determining the failure probabilities of the nodes through collecting additional case data and field monitoring to reduce the impact of subjective factors.

## Supporting information

S1 Text (DOCX)
