# Supplementary material for: Analysis of collapse risks under cut and cover method based on multi-state fuzzy Bayesian network
Source: PLoS One. 2025 May 7;20(5):e0321382. doi: 10.1371/journal.pone.0321382 (PMC12058190; doi:10.1371/journal.pone.0321382)
Supplement: S1 Text — (DOCX) [file pone.0321382.s001.docx]

**Supplementary Materials**

**Appendix A – Accident data**

Statistical Cases of Collapse Accidents in Subway Station Construction Using the Cut and Cover Method

| Serial Number | Occurrence Time | Location | Type of Collapse Accident | Casualties | Main Reasons |
| --- | --- | --- | --- | --- | --- |
| 1 | 2006.01.03 | Beijing | Sudden water gushing, ground collapse | No casualties | Underground pipeline leakage, inadequate investigation |
| 2 | 2006.06.26 | Beijing | Shaft construction collapse | 2 deaths | Subsurface quicksand, natural factors, and inadequate investigation |
| 3 | 2006.08.02 | Guangzhou | Soil collapse during construction | 2 deaths | Sudden collapse of weak soil layer |
| 4 | 2007.01.17 | Guangzhou | Ground subsidence | No casualties | Imbalanced water and soil pressure |
| 5 | 2007.02.05 | Nanjing | Ground subsidence due to pipeline seepage | Gas pipeline explosion, no casualties | Long-term aging of soil layers at the collapsed section, pipeline leakage |
| 6 | 2007.03.28 | Beijing | Tunnel entrance collapse accident at the subway station | 6 deaths | Lack of specific geological conditions, lack of emergency rescue measures |
| 7 | 2007.05.28 | Nanjing | Landslide at the subway station | 2 deaths | After being soaked by rainwater, silty soil slope experienced an accident |
| 8 | 2007.10.01 | Guangzhou | Arch top gravel layer seepage and subsequent collapse | No casualties | Seepage and collapse after arch top gravel layer was saturated with water |
| 9 | 2007.11.20 | Nanjing | Ground subsidence | No casualties | Local strata released void gases, forming groundwater channels leading to water and sand gushing phenomena |
| 10 | 2008.01.17 | Guangzhou | Road surface collapse due to sudden water gushing during construction | No casualties | Sudden water gushing during construction |
| 11 | 2008.03.10 | Shenzhen | Foundation pit collapse | No casualties | Over-excavation beyond standard height, slow installation speed of support structures |
| 12 | 2008.04.01 | Shenzhen | Formwork collapse | 3 deaths, 2 injuries | Small load-bearing capacity of bolts used to reinforce column formwork led to collapse |
| 13 | 2008.11.15 | Hangzhou | Foundation pit collapse | 21 deaths, 24 injuries | Illegal construction practices, weak support systems, failure of construction monitoring |
| 14 | 2009.08.02 | Xi'an | Foundation pit collapse | 2 deaths | Continuous rainfall, lack of timely excavation and support measures |
| 15 | 2010.10.22 | Guangzhou | Road surface subsidence | No casualties | Complex geological environment, abundant groundwater |
| 16 | 2010.11.23 | Hangzhou | Foundation pit collapse | 1 death | Soil and rock slide |
| 17 | 2011.05.06 | Tianjin | Ground subsidence | No casualties | Complex geological environment, sudden mud and water gushing |
| 18 | 2012.11.09 | Nanchang | Construction collapse accident | 1 death | Collapse caused by removal of retaining structure anchor rods |
| 19 | 2012.12.31 | Shanghai | Construction collapse accident | 5 deaths, 18 injuries | Improper setup of steel frame structure led to partial instability of support system |
| 20 | 2013.01.28 | Guangzhou | Surface collapse | No casualties | The construction method used caused the upper thin rock layer to collapse |
| 21 | 2013.01.30 | Nanjing | Foundation pit collapse | No casualties | Sewer pipe explosion and cracking |
| 22 | 2013.02.22 | Zhengzhou | Collapse accident | 2 deaths | Excavation of foundation pit caused soil instability |
| 23 | 2013.04.24 | Lanzhou | Collapse accident | 2 deaths | Violations during formwork removal by construction technicians |
| 24 | 2013.05.06 | Xi'an | Foundation pit collapse | 5 deaths | Natural factors, rain causing soil loosening |
| 25 | 2013.06.13 | Changchun | Foundation pit collapse | 1 death | Local detachment of steel supports caused lateral sliding of retaining structures |
| 26 | 2013.08.19 | Ningbo | Foundation pit collapse | 2 deaths, 2 injuries | Natural factors (heavy rainfall), support instability |
| 27 | 2014.03.02 | Lanzhou | Foundation pit collapse | 1 death, 1 injury | Soil water gushing |
| 28 | 2014.03.23 | Beijing | Collapse accident | 1 death | Complex geological environment, large piles of earth collapsed onto tunnel top iron frames |
| 29 | 2014.04.24 | Hangzhou | Road surface subsidence | No casualties | Heat generated during construction melted soil layers, leading to water and soil loss |
| 30 | 2014.04.28 | Lanzhou | Collapse accident | 2 deaths | Pipeline relocation process resulted in collapse |
| 31 | 2014.06.07 | Wuhan | Collapse accident | No casualties but surrounding buildings showed settlement and cracks | Complex geological environment, sudden water and sand gushing |
| 32 | 2014.08.11 | Guiyang | Collapse accident | 1 death | Collapse occurred during civil engineering construction |
| 33 | 2014.09.07 | Beijing | Road surface subsidence | No casualties | Pipeline rupture |
| 34 | 2014.10.07 | Nanning | Collapse accident | 1 death, 2 missing | Natural factors, complex geological environment, human and management factors |
| 35 | 2014.12.17 | Nanjing | Collapse accident | 4 deaths, 3 injuries | Reinforcement collapse, support system instability |
| 36 | 2015.02.06 | Qingdao | Foundation pit collapse | 1 death | Internal collapse within the foundation pit |
| 37 | 2015.03.29 | Tianjin | Collapse accident | No casualties | Sudden water penetration |
| 38 | 2015.05.11 | Shenyang | Foundation pit collapse | 1 death | During excavation of foundation pit encountered pipelines, no emergency management measures were established |
| 39 | 2015.05.13 | Nanning | Collapse accident | 1 death | Soil and stone collapse due to violation of operating procedures by construction workers |
| 40 | 2015.05.17 | Guiyang | Collapse accident | 1 death | Issues with construction safety management |
| 41 | 2015.11.16 | Nanjing | Ground subsidence | No casualties | Abundant underground water, collapse occurred during drainage |
| 42 | 2016.03.05 | Shenzhen | Collapse accident | 1 death, 1 injury | Direct cause: poor adhesion between diaphragm wall upper part and road surface, strong permeability; heavy vehicles on the road caused soil instability. Indirect cause: inadequate identification of safety hazards |
| 43 | 2016.05.14 | Nanchang | Road surface subsidence | No casualties | Pipeline rupture causing sand gushing |
| 44 | 2016.07.08 | Hangzhou | Foundation pit collapse | 4 deaths | Increased groundwater levels weakened soil strength, increased risk of water and sand gushing |
| 45 | 2016.07.29 | Chongqing | Collapse accident | 3 deaths | Premature removal of temporary supports before they were fully stabilized without setting longitudinal temporary supports |
| 46 | 2016.10.19 | Shenyang | Collapse accident | 3 deaths | Natural factors (prolonged heavy rainfall) |
| 47 | 2017.04.19 | Shenzhen | Construction collapse | 1 death, 3 injuries | Collapse of steel reinforcement framework |
| 48 | 2017.05.11 | Shenzhen | Soil collapse | 3 deaths, 1 injury | Direct cause: over-excavation of foundation pit, overload on slope top, natural factors. Indirect cause: ineffective supervision by supervisory party, unaddressed safety hazards |
| 49 | 2018.01.26 | Guangzhou | Tunnel collapse | 3 deaths | Natural factors (complex geological environment) |
| 50 | 2018.02.07 | Foshan | Ground collapse | 11 deaths, 8 injuries | Sudden water gushing incident |
| 51 | 2018.08.08 | Guiyang | Collapse accident | 3 deaths | Secondary lining steel bar collapse |
| 52 | 2019.05.27 | Qingdao | Construction collapse | 5 deaths | Soft soil layer construction leading to road surface settlement |
| 53 | 2019.12.01 | Guangzhou | Ground collapse | 3 people trapped | Natural factors (complex geological environment), arch top water penetration during construction |
| 54 | 2021.05.07 | Hangzhou | Soil collapse | 1 death | Intensive construction activities on the slope top before initial lining formed, resulting in overload on slope top |
| 55 | 2021.07.20 | Zhengzhou | Construction site collapse | No casualties yet reported | Natural factors |
| 56 | 2021.09.10 | Chengdu | Steel structure collapse | 4 deaths, 18 injuries | Shed net frame collapse while setting up ground dust reduction and noise reduction sheds |

#

**Appendix B – Study Survey**

Subway Station Cut and Cover Construction Collapse

Accident Survey

Dear Esteemed Expert,

I sincerely appreciate your valuable time in participating in this survey. I am a researcher affiliated with Lanzhou University of Technology, Gansu Province, conducting this study to better understand the underlying mechanisms contributing to collapse accidents during open-cut construction for subway stations. Your insights are crucial for gathering necessary data that will support my research endeavors.

This questionnaire is divided into two main sections: The first section requests your background information, which will help contextualize the data collected. The second section focuses on assessing the likelihood of various causal factors leading to collapse incidents specifically within the context of open-cut subway station construction. Please be assured that all responses will be treated confidentially and used solely for academic purposes, ensuring no negative impact or inconvenience to you.

Thank you once again for your invaluable cooperation and contribution to advancing our understanding in this field.

**Part One: Basic Information**

**Background Information**

1. What is your years of work experience? 【Single Choice Question】

○ 2年及以下Less than 2 years

○ 2~5年2 ~ 5 years

○ 6~10年6 ~9 years

○ 10年及以上10 years and above

2. What is your age? 【Single Choice Question】

○ Below 30 years old

○ 30~39岁30 ~ 39 years old

○ 40~49岁40 ~ 49 years old

○ 50岁及以上50 years old and above

3. What is your highest level of education (including currently enrolled)? 【Single Choice Question】

○ High School and below

○ College Graduate

○ Bachelor's degree

○ Postgraduate and above

4. Your Position? 【Single Choice Question】

○ Project Manager

○ Head of Department

○ Project Manager

○ Other managers

**Part Two: Investigation on the Likelihood of Causal Factors Leading to Subway Station Construction Collapse Accidents**

5. Please score the following items 【Scoring Question】 (Please fill in a number between 1-7 for scoring)

Here are the accident phenomena that are likely to occur during the construction process of subway stations. The numbers 1-7 represent: "Very low possibility", "Low possibility", "Nearly low possibility", "Moderate possibility", "Nearly high possibility", "High possibility", "Very high possibility". Please select according to the likelihood of each event occurring.

**Survey on the Likelihood of Causal Factors Leading to Collapse Accidents During Subway Station**

**Cut and Cover Method Construction**

| Number | Accident Phenomenon | Possibility of Factor Occurrence | | | | |  |  |
| --- | --- | --- | --- | --- | --- | --- | --- | --- |
|  |  | 1 | 2 | 3 | 4 | 5 | 6 | 7 |
| 1 | Improper installation of horizontal supports X_1_ | □ | □ | □ | □ | □ | □ | □ |
| 2 | Inadequate strength of support structures X_2_ | □ | □ | □ | □ | □ | □ | □ |
| 3 | Improper arrangement of support systems X_3_ | □ | □ | □ | □ | □ | □ | □ |
| 4 | Improper excavation methods X_4_ | □ | □ | □ | □ | □ | □ | □ |
| 5 | Overloading or excessive vibrations of surrounding soil X_5_ | □ | □ | □ | □ | □ | □ | □ |
| 6 | Excessive slope steepness X_6_ | □ | □ | □ | □ | □ | □ | □ |
| 7 | Long-term rainfall X_7_ | □ | □ | □ | □ | □ | □ | □ |
| 8 | Adverse geological and hydrological conditions X_8_ | □ | □ | □ | □ | □ | □ | □ |
| 9 | Soil erosion X_9_ | □ | □ | □ | □ | □ | □ | □ |
| 10 | Collapse above pipelines X_10_ | □ | □ | □ | □ | □ | □ | □ |
| 11 | Pipeline leakage or damage X_11_ | □ | □ | □ | □ | □ | □ | □ |
| 12 | Excessive over-excavation or under-excavation X_12_ | □ | □ | □ | □ | □ | □ | □ |
| 13 | Seepage in gravel layers at arch crowns X_13_ | □ | □ | □ | □ | □ | □ | □ |
| 14 | Improper drainage methods X_14_ | □ | □ | □ | □ | □ | □ | □ |
| 15 | Local formation water inrush and sand inflow X_15_ | □ | □ | □ | □ | □ | □ | □ |
| 16 | Water inflow at the bottom of diaphragm walls X_16_ | □ | □ | □ | □ | □ | □ | □ |
